# Establishing a diagnostic scale of subacute thyroiditis without radioisotope scanning

**DOI:** 10.1186/s12902-020-00554-z

**Published:** 2020-05-27

**Authors:** Zhouyi Xiong, Chunying Luo, Li Wang, Bin Xiong, Jianneng Wu

**Affiliations:** Department of Endocrinology, Yuebei People’s Hospital, NO. 133 Huimin South Road, Shaoguan, Guangdong Province China

**Keywords:** Subacute thyroiditis, Radioisotope scanning, Diagnostic scale, Logistic regression analysis

## Abstract

**Background:**

Radioisotope scanning is important to diagnose subacute thyroiditis (SAT), but it’s not always available. So we aim to establish a diagnostic scale for SAT without radioisotope scanning.

**Methods:**

The suspected SAT patients hospitalized in Yuebei people’s Hospital from January 2012 to December 2016 were selected and divided into study group and control group according to whether they were diagnosed as SAT. The clinical indexes of two groups were collected and the diagnostic scale of SAT was established by using binary logistic regression analysis. The effectiveness of the scale was evaluated by ROC curve.

**Results:**

Of 309 patients, 58.25% of patients were confirmed with SAT and the remaining 41.75% of patients were not diagnosed with SAT. After univariate analysis, variables which were considered statistically different(*P* < 0. 05) between the two groups were selected as independent variables and the diagnosis of SAT was taken as dependent variable in the binary logistic regression model. The logistic regression model consisted of 4 variables, each was thyroid tenderness, firm on palpation, increased ESR and elevated thyroid hormone level. The *P* value of Omnibus tests was≤0. 001 and the Nagelkerke R Square was 0. 915. The diagnostic scoring scale was established with these four variables according to their regression coefficient. The area under the ROC curve for this diagnostic scale was 0. 991(95% confidence interval, 0. 982–0.999). The highest Youden index was 0. 912, the corresponding cut-off point was 7. Internally validation shows a sensitivity of 92. 78% and a specificity of 98.45% of our scale.

**Conclusions:**

We established and validated a diagnostic scale for SAT without the need for radioisotope scanning for the first time. It has good application in institutions that do not have radioisotope machines or among pregnant and lactating women.

## Background

Subacute thyroiditis (SAT), also known as de Quervain’s thyroiditis, is a self-limited disease of the thyroid gland. It is the most common disease with thyroid pain and the incidence is reported to be 3.6 cases in every 100, 000 people [1], but it is elevated in China as we have observed. The diagnosis of SAT is mainly based on thyroid pain, increased erythrocyte sedimentation rate (ESR) or C-reactive protein (CRP), and most importantly, high serum thyroid hormone concentrations while the uptake of radioactive iodine or technetium is low (because of the destruction of thyrocytes). However, the radioisotope scanning is not always available in primary care institutions, and sometimes it is contraindicated in specific situations such as in pregnant or lactating women. Thus, some patients might be misdiagnosed as Graves’ disease [2], upper respiratory infection, dental problem [3], pharyngitis or abscess. Wrong diagnoses seriously affect the treatment and prognosis of this disease. An alternative method for diagnosing SAT is needed in clinical practice. Thus, the purpose of this study is to establish a SAT diagnostic scale without radioisotope scanning and using simple clinical indicators.

## Methods

We performed a retrospective study among adult patients who were suspected to have SAT and were also accepted 99 m-Tc thyroid static imaging in Yuebei People’s Hospital. Inclusion criteria: Patients with anterior neck pain, enlarged thyroid or thyrotoxicosis as the chief complaint for the first time and were admitted to hospital between January 2012 to December 2016. All patients received no treatment before and each one of them have accepted 99 m-Tc thyroid static imaging for diagnosing after being admitted. Exclusion criteria: Patients who were previously confirmed with Graves’ disease, thyroid tumor, acute suppurative thyroidits, Hashimoto’s thyroiditis or any other thyroid diseases. We also excluded confirmed upper respiratory infection, dental disease, pregnancy or recurrent SAT. The medical records of all patients were thoroughly examined, and the final diagnoses were reassessed by endocrinologists. Diagnosis of SAT were based on clinical manifestations and laboratory test results, including one or both side neck pain, thyroid swelling and/or thyroid tenderness, increased ESR and/or CRP, elevated serum thyroid hormone concentrations and suppressed uptake of Technetium-99 m [4]. Sometimes not every patient could meet all the above standard, the final diagnosis was made by at least two endocrinologists under these circumstances. Patients who were confirmed with SAT formed the study group and those who were confirmed with not SAT formed the control group. The study was approved by the hospital Ethics Review Committee. All patients have signed informed consent.

All variables were extracted from the database of the hospital. We collected the vital signs and physical examination results of all patients during the period of first evaluation. We also collected the results of laboratory tests and 99 m-Tc scan results of patients before diagnosing and treatment. Serum levels of TT3, TT4, FT3, FT4, TSH, TPOAb and TgAb were measured using commercial kits provided by Roche Diagnostics, Indianapolis, IN. The corresponding reference ranges of serum TT3, TT4, FT3, FT4, THS, TPOAb and TgAb were 1.3–3.1 nmol/l, 66–181 nmol/l, 3.1–6.8 pmol/l, 12–22 pmol/l, 0.27–4.2 μIU/ml, 0–34 IU/ml and 0–155 IU/ml, respectively. Missing values were also counted. Descriptive data were shown as mean ± SD (for parametric tests) or frequencies (for nonparametric tests). We performed univariate analysis of all variables between groups. Student’s *t* test was used if continuous variables were subject to normal distribution or satisfying homogeneity of variance. If not, Wilcoxon’s rank sum test was used. For dichotomous variables, Chi-square test was used. All variables with *P*<0. 05 in the univariate analysis were considered statistically different and were selected as covariates in the full multivariable logistic regression model with the diagnosis of SAT as the dependent variable.

We used the *forward:likelihood ratio* method to generate the statistically optimal logistic regression model (with *entry* as *P* ≤ 0. 05 and *removal* as *P*>0. 10). Meanwhile, we carried out the omnibus tests of model coefficients and calculated the Nagelkerke R Square. Then, for clinical use, we developed a diagnostic scale with the use of variables in the regression model, with weighting based on each regression coefficient. According to our clinical scale, each patients was assigned a score. We used these scores to draw a receiver operating characteristic (ROC) curve to evaluate the diagnostic performance of our scale. The optimal cut-off point was determined by Youden index (Youden index equals to sensitivity plus specificity minus 100%). All patients were once again diagnosed using our diagnostic scale,and were compared with former diagnosis to evaluate the diagnostic test characteristics (sensitivity, specificity, positive and negative predictive values, false positive rate, false negative rate and accuracy) using 2 by 2 tables.

Statistical analyses were performed using IBM SPSS Statistics for Windows, version 25. 0 (IBM Corp., Armonk, NY, USA). *P*<0. 05 was considered statistically significant.

## Results

We included 309 inpatients in our study. Among them, 180 patients (58.25%) were confirmed with SAT and 129 patients (41.75%) were diagnosed with other diseases such as nodular goiter (55 patients), Graves’ disease (50 patients), thyroid tumor (7 patients), Hashimoto’s thyroiditis (8 patients), upper respiratory infection (1 patient) and other diseases (8 patients). The mean age of the SAT group was 46. 77 ± 10. 30 years, and 82. 22% were females. The mean age of the control group was 46.46 ± 14.17 years, and 75.19% were females. For 89. 14% patients of the SAT group, the thyroid was not visualized or was visualized poorly on 99 m-Tc thyroid static imaging. Table 1 shows the descriptive statistics and univariate analysis of clinical variables between the two groups.

**Table 1:** Descriptive statistics and univariate analysis of clinical variables between the two groups

Variables	Study group	Control group	*P* value
Male sex, No. (%)	32 (17. 78)	32 (24. 81)	0. 133
Age (years)	46. 77 ± 10. 30	44. 46 ± 14. 17	0. 828
Height (cm)	158. 18 ± 6. 43	157. 89 ± 7. 42	0. 751
Weight (kg)	55. 01 ± 8. 81	53. 67 ± 9. 65	0. 106
Systolic pressure (mmHg)	118. 69 ± 13. 40	123. 14 ± 16. 78	0. 019
Diastolic pressure (mmHg)	76. 07 ± 9. 52	77. 63 ± 11. 10	0. 278
Heart rate (bpm)	87. 42 ± 12. 90	86. 93 ± 16. 02	0. 380
Elevated heart rate, No. (%)	21 (11. 77)	27 (20. 93)	0. 028
Prior upper respiratory infection, No. (%)	26 (14. 44)	4 (3. 10)	0. 001
Fever, No. (%)	60 (33. 33)	0	≤0. 001
Neck pain, No. (%)	174 (96. 67)	21 (16. 28)	≤0. 001
Thyroid tenderness, No. (%)	176 (97. 78)	9 (6. 98)	≤0. 001
Odynophagia, No. (%)	135 (75. 00)	6 (4. 65)	≤0. 001
Radiating pain, No. (%)	89 (49. 44)	5 (3. 88)	≤0. 001
Palpitation, No. (%)	41 (22. 78)	47 (36. 46)	0. 009
Hands tremble, No. (%)	10 (5. 56)	28 (21. 71)	≤0. 001
Weight loss, No. (%)	26 (14. 44)	33 (25. 58)	0. 014
Thyroid swelling, No. (%)	168 (93. 33)	114 (83. 37)	0. 128
Firm on palpation, No. (%)	148 (82. 22)	8 (6. 20)	≤0. 001
Elevated white blood cell, No. (%)	45 (25. 00)	4 (3. 10)	≤0. 001
Elevated neutrophil, No. (%)	65 (36. 11)	8 (6. 20)	≤0. 001
Increased ESR, No. (%)	140 (77. 78)	5 (3. 88)	≤0. 001
Elevated CRP, No. (%)	127 (70. 56)	9 (6. 98)	≤0. 001
Elevated thyroid hormone level, No. (%)	106 (58. 89)	57 (44. 19)	0. 011
TT3(nmol/L)	2. 69 ± 1. 38	3. 09 ± 2. 79	0. 087
TT4(nmol/L)	165. 27 ± 61. 09	137. 77 ± 74. 63	≤0. 001
FT3(pmol/L)	9. 34 ± 8. 94	15. 20 ± 14. 87	0. 877
FT4(pmol/L)	31. 01 ± 19. 38	40. 63 ± 34. 00	0. 703
TSH (μIU/ml)	0. 51 ± 1. 40	2. 89 ± 12. 45	0. 168
TPOAb (IU/ml)	34. 56 ± 67. 14	132. 53 ± 185. 56	0. 026
TgAb (IU/ml)	283. 15 ± 534. 56	366. 74 ± 794. 74	0. 046
Suppressed uptake of Technetium-99 m, No. (%)	156 (89. 14)	19 (14. 73)	≤0. 001
FT3/FT4	0. 307 ± 0. 20	0. 34 ± 0. 10	≤0. 001

Descriptive data was shown as mean ± SD or frequencies(%). The corresponding reference ranges of serum TT3, TT4, FT3, FT4, THS, TPOAb and TgAb were 1.3–3.1 nmol/l, 66–181 nmol/l, 3.1–6.8 pmol/l, 12–22 pmol/l, 0.27–4.2 μIU/ml, 0–34 IU/ml and 0–155 IU/ml, respectively

After univariate analysis, systolic pressure, elevated heart rate, prior upper respiratory infection, fever, neck pain, thyroid tenderness, odynophagia, radiating pain, palpitation, weight loss, firm on palpation, elevated white blood cell, elevated neutrophi, increased ESR, elevated CRP, elevated thyroid hormone level, FT3 to FT4 ratio and suppressed uptake of Technetium-99 m were considered significantly different between the two groups and were selected as covariates in the full multivariable logistic regression model. Because the suppressed uptake of Technetium-99 m was the diagnostic method our study wanted to exclude, this variable was eliminated in the full multivariable logistic regression model artificially. The logistic regression model finally consisted of 4 variables that were independently predictive factors (as shown in Table 2) with the diagnosis of SAT as the dependent variable. The equation for the logistic model was as follows:

**Table 2:** Variables of the final logistic regression model and clinical scores of the diagnostic scale

Variable	Regression coeffient	OR(95%CI)	*P* value	Clinical score
Thyroid tenderness	5. 801	330.628 (39.529–2765.422)	≤0. 001	6
Firm on palpation	2. 586	13.270 (2.720–64.731)	0. 001	3
Increased ESR	2. 520	12.430 (2.249–68.709)	0. 004	3
elevated thyroid hormone level	2. 073	7.949 (0.932–67.782)	0. 058	2
Maximum score				14

The *P* value of Omnibus tests was ≤0. 001 and the Nagelkerke R Square was 0. 915.

*P* = -5.515(constant) + (5.801*thyroid tenderness) + (2. 586*firm on palpation) + (2. 520*increased ESR) + (2. 073* elevated thyroid hormone level).

**Fig. 1:**
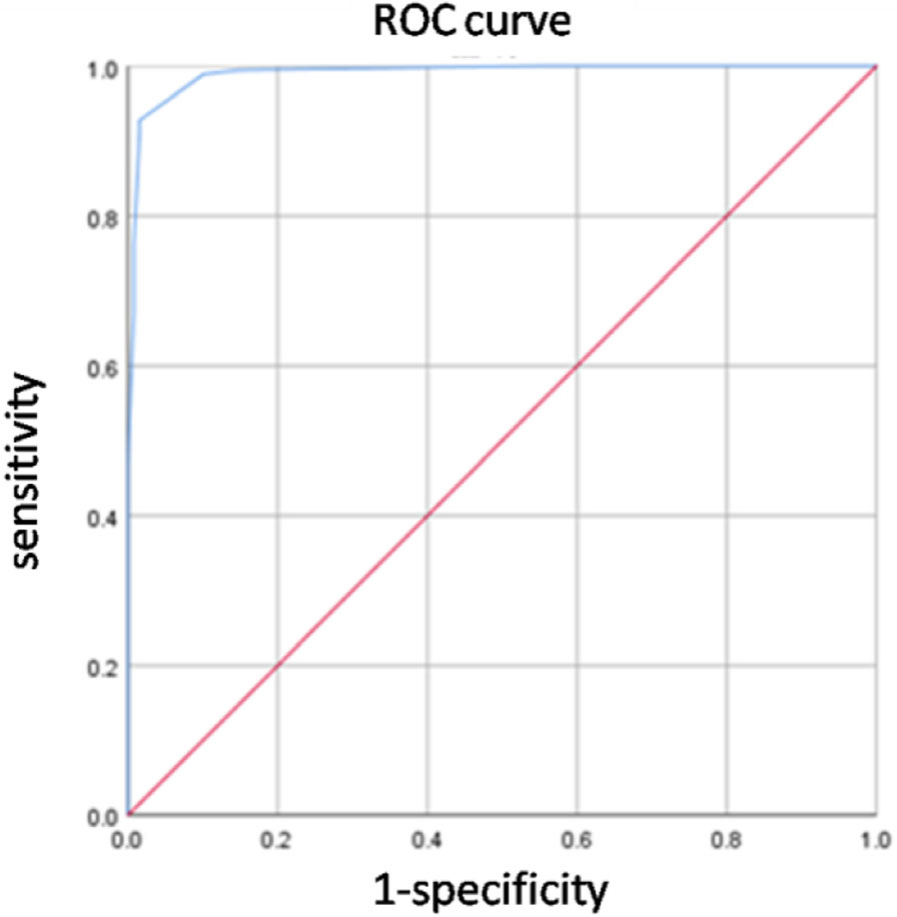
Receiver operating characteristic (ROC) curve shows the discriminative performance of the diagnostic scale. Discriminative performance is the ability of the scale to distinguish between patients with and without subacute thyroiditis. The ROC curve plots the sensitivity vs 1-specificity for different cut-off values of the scale. The diagonal line indicates the curve for a virtual model without predicting value (ROC of 0. 5)

The regression coefficients of these 4 variables were then rescaled to easy-to-use scores (as shown in Table 2), with the total points being 14. Then we performed ROC curve analysis of the diagnostic scale. The area under the ROC curve was 0. 991(95% confidence interval, 0. 982–0.999) as shown in Fig. 1. The highest Youden index was 0. 912, while the corresponding cut-off point was 7.

With the optimal cut-off point, all patients were once again diagnosed and compared with former diagnosis to evaluate the diagnostic test characteristics using 2 by 2 tables (as shown in Table 3). Our scale has a sensitivity of 92.78% and a specificity of 98.45%. Positive and negative predictive values were 98. 82 and 90.71%, respectively. False positive and false negative rates were 1.55 and 7.22% respectively. The accuracy of our diagnostic scale was 95.15%.

**Table 3:** Diagnostic test results compared with former diagnosis (reference test) using 2 by 2 tables

		Reference test		
		SAT	No SAT	
Test results	SAT	a 167	b 2	a + b = 169
	No SAT	c 13	d 127	c + d = 140
		a + c = 180	b + d = 129	

a, true positive; b, false positive; c, false negative; d, true negative.

Sensitivity = a/(a + c) × 100% = 92.78%; specificity = d/(b + d) × 100% = 98.45%; positive predictive value = a/(a + b) × 100% = 98. 82%; negative predictive value = d/(c + d) × 100% = 90.71%; False positive rate = b/(b + d) × 100% = 1.55%; false negative rate = c/(a + c) × 100% = 7.22%; accuracy = (a + d)/n × 100% = 95.15%.

## Disccusion

Subacute thyroiditis is a non-bacterial inflammatory disease of the thyroid gland. It comprises nearly 3–6% of all thyroid lesions [5]. The diagnosis is made by a combination of clinical manifestations, physical examinations and laboratory tests [6]. Tissue diagnosis is not a routine, but only necessary in rare cases such as in differential diagnosis of thyroid cancer [7]. Usually, the typical SAT has three stages: thyrotoxicosis stage, hypothyroid stage and normal thyroid function stage. However, patients may come to hospital at any stage, and the clinical manifestations may not show in a typical way, which makes the diagnosis more difficult. Furthermore, a study showed most laboratory results associated with thyrotoxicosis have reached abnormal levels within 3 weeks after onset. But longer time-lags could existed between the onset of clinical symptoms and the appearance of abnormal laboratory findings in patients with SAT [8]. Therefore, elevated thyroid hormone level and suppressed uptake of Technetium-99 m (99 m-Tc) or 131I at the same time play a significant role in diagnosing. However, the radioisotope scanning is not always available in every hospital and sometimes it is contraindicated in specific situations such as in pregnant or lactating women. So we developed and internally validated a diagnostic scale for SAT without the need for radioisotope scanning.

Our scale contained a limited number of signs and symptoms and 2 laboratory tests, which were easy to perform in primary care institutions. Thyroid tenderness will appear almost 100% due to the inflammatory destruction of thyrocytes. In our diagnostic scale, the regression coefficient of this variable was 5. 801 with a clinical score of 6, demonstrating the importance of thyroid tenderness in diagnosing SAT. Follicular epithelial cells and multinuclear giant cells against a dirty background is the pathological characteristics of SAT, resulting in feeling firm on thyroid palpation [9]. The variable of “firm on palpation” obtained a clinical score of 3 according to the regression coefficient in our scale. Part of the reason is that the less inflammatory cell infiltration can lead to the less firm thyroid on palpation. ESR is a sensitive indicator of acute inflammation and always elevated in SAT patients [4, 6]. But ESR is not a special feature to some certain diseases, so it earned only 3 scores in our scale. In the first stage of SAT, all patients are tested as elevated thyroid hormone level, which is defined by elevated FT3 and/or FT4 concentrations and/or suppressed TSH level in our study, whether they have symptoms or not. However, patients may be at other stages when they come to hospital. Their thyroid function may be normal or even decreased. So the variable of elevated thyroid hormone level was assigned 2 scores in our scale. The *P* value of Omnibus tests of our model was ≤0. 001 and the Nagelkerke R Square was 0. 915, demonstrating that our model has statistically significant differences and better goodness of fit. According to the ROC curve analysis, the optimal cut-off point was 7. At this point, our scale has high sensitivity (92.78%) and specificity (98. 45%) in recruited patients. The accuracy of our diagnostic scale was also high (95. 15%). To our knowledge, this diagnostic study is the first one to develop a clinical prediction model for the diagnosis of SAT.

A lot of studies have shown that the etiology of SAT was related to viral infection such as coxsackievirus, echovirus, mumps, measles, influenza and other viruses [10, 11] because there was a flu-like syndrome before the disease onset. In our study, only 14. 44% patients had upper respiratory infection before SAT, maybe due to the blurry memory of patients and the inapparent infections. Espinoza et al. [12] have compared the diagnostic value of radioactive iodine uptake, 99 m-Tc thyroid static imaging and thyroid ultrasonography. They found both radioisotope scanning had a better correlation with the clinical diagnosis of SAT than that with thyroid ultrasonography. In our study, we used the 99 m-Tc thyroid static imaging to evaluate the thyroid uptake function instead of radioactive iodine uptake because radioactive iodine uptake needs more time (24 h) and is more complicated to operate. As reported by Frates et al. [13], the typical appearance of sonography of SAT was a patchy, poorly defined hypoechoic process that could affect a portion of one or both lobes, an entire lobe, or the entire gland. However, they also found that the sonographic findings of SAT could mimic a large nodule replacing the lobe, the changes of lymphocytic, Hashimoto’s thyroiditis, thyroid carcinoma or thyroid lymphoma, leading to the differential diagnosis became more difficult. Furthermore, sonography is a relatively subjective examination and requires experienced doctors to get analysable results. So we excluded sonography as a variable from our model. Fever is also a clinical presentation of SAT. Sometimes fever was the only clinical manifestation as reported by Dalugama [14]. But this situation is rare and fever can present in many diseases. So fever, though there was significant difference between the two groups, was not included in our regression model. Some studies have demonstrated that a higher ratio of FT3 to FT4 supported a diagnosis of Graves’ disease and a very low ratio supported a diagnosis of SAT [15, 16]. In our study, the ratio of FT3 to FT4 showed significant difference between the two groups, indicating its diagnostic value. But it’s not included in the final regression model. The reason is that the control group was not only consisted of Graves’ disease patients, the diagonsitic value of this ratio is limited.

Our study has some limitations. First, it was a retrospective study. We could only collected data from the hospital system other than examining the patients by ourselves, which may cause the inconsistency. Second, we have excluded patients who did not perform radioisotope scanning or in pregnant or lactating women, which might lead to a selection bias. Third, our patients were all at their first attack and first visit to hospital. We were not sure if our diagnostic model was suitable for recurrent patients or treated patients. Fourth, it’s difficult to differentiate acute thyroiditis and subacute thyroiditis according to our scale. To prevent misdiagnosis, we recommend using B-ultrasound, FNC and pathogenic culture to distinguish this two diseases at this circumstance. The last but not the least, the specificity and sensitivity of our diagnostic scale were really high because we only did internal validation and obtained overoptimistic results. Therefore, external validation is needed before wider application.

## Conclusions

In conclusion, we established a diagnostic scale for SAT without the need for radioisotope scanning for the first time. It is an excellent and easy way to diagnose SAT. Although it cannot fully replace radioisotope scanning, it has good application in institutions that have no access to radioisotope machines or in pregnant and lactating women.

## Data Availability

The datasets used during the current study are available from the corresponding author on reasonable request.

## Abbreviations

SAT: Subacute thyroiditis
ESR: Erythrocyte sedimentation rate
CRP: C-reactive protein
ROC: Receiver operating characteristic
TT3: Total triiodothyronine
TT4: Total thyroxine
FT3: Free triiodothyronine
FT4: Free thyroxine
TSH: Thyroid stimulating hormone
TPOAb: Thyroid peroxidase antibodies
TgAb: Thyroglobulin antibodies
99 m-Tc: Technetium-99 m
